# Cyanomethyl Vinyl Ethers Against *Naegleria fowleri*

**DOI:** 10.1021/acschemneuro.3c00110

**Published:** 2023-05-11

**Authors:** Javier Chao-Pellicer, Iñigo Arberas-Jiménez, Samuel Delgado-Hernández, Ines Sifaoui, David Tejedor, Fernando García-Tellado, José E. Piñero, Jacob Lorenzo-Morales

**Affiliations:** † Instituto Universitario de Enfermedades Tropicales y Salud Pública de Canarias, Universidad de La Laguna, Avda. Astrofísico Fco. Sánchez, S/N, La Laguna 38203, Tenerife, Islas Canarias, Spain; ‡ Departamento de Obstetricia y Ginecología, Pediatría, Medicina Preventiva y Salud Pública, Toxicología, Medicina Legal y Forense y Parasitología, Universidad de La Laguna, Tenerife, Islas Canarias 38200, Spain; § Centro de Investigación Biomédica en Red de Enfermedades Infecciosas (CIBERINFEC), Instituto de Salud Carlos III, Madrid 28220, Spain; ∥ Instituto de Productos Naturales y Agrobiología, Consejo Superior de Investigaciones Científicas, Avda. Fco. Sánchez 3, La Laguna 38206, Tenerife, Islas Canarias, Spain; ⊥ Departamento de Química. Unidad Departamental de Química Analítica, Universidad de La Laguna (ULL), Tenerife 38206, Spain

**Keywords:** Naegleria fowleri, primary amoebic meningoencephalitis, cyanomethyl vinyl ethers, cytotoxicity, apoptosis, programmed cell death

## Abstract

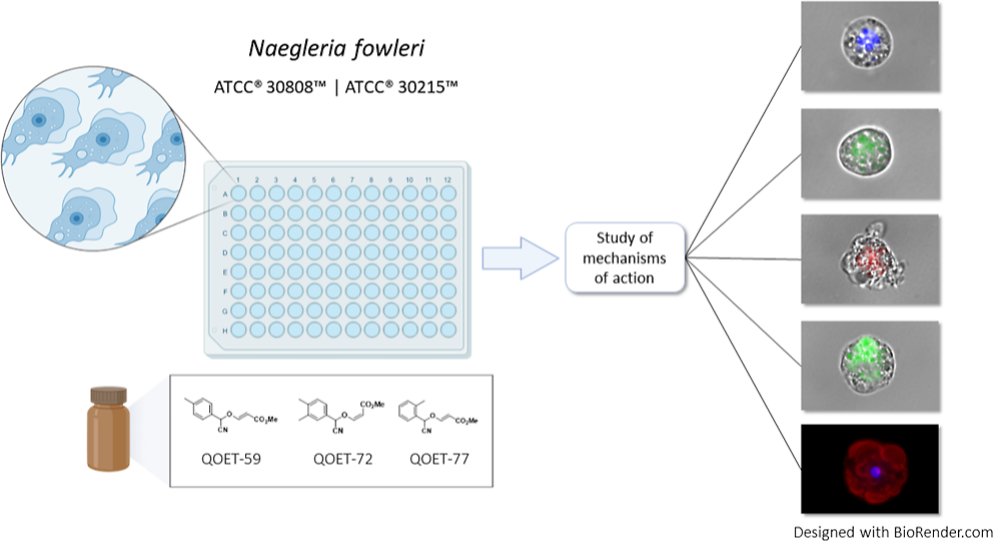

*Naegleria fowleri* is a pathogenic amoeba that causes a fulminant and rapidly progressive disease affecting the central nervous system called primary amoebic meningoencephalitis (PAM). Moreover, the disease is fatal in more than 97% of the reported cases, mostly affecting children and young people after practicing aquatic activities in nontreated fresh and warm water bodies contaminated with these amoebae. Currently, the treatment of primary amoebic meningoencephalitis is based on a combination of different antibiotics and antifungals, which are not entirely effective and lead to numerous side effects. In the recent years, research against PAM is focused on the search of novel, less toxic, and fully effective antiamoebic agents. Previous studies have reported the activity of cyano-substituted molecules in different protozoa. Therefore, the activity of 46 novel synthetic cyanomethyl vinyl ethers (QOET-51 to QOET-96) against two type strains of *N. fowleri* (ATCC 30808 and ATCC 30215) was determined. The data showed that QOET-51, QOET-59, QOET-64, QOET-67, QOET-72, QOET-77, and QOET-79 were the most active molecules. In fact, the selectivity index (CC_50_/IC_50_) was sixfold higher when compared to the activities of the drugs of reference. In addition, the mechanism of action of these compounds was studied, with the aim to demonstrate the induction of a programmed cell death process in *N. fowleri*.

## Introduction

*Naegleria fowleri*, commonly known as the “brain-eating amoeba,” is a free-living amoeba,^1^ which is able to inhabit warm water bodies where the temperature is deregulated and chlorination is minimal.^2−4^ In addition, this amoebic species is able to infect the brain and cause a disease known as primary amoebic meningoencephalitis (PAM).^5^

This rare disease that affects the central nervous system (CNS) results in the death of more than 95% of the described cases.^6−9^ Even though just over 400 cases of the disease have been reported worldwide, climate change and increase awareness could be the factors of higher infection rates.^10−13^ However, a better idea of the incidence is given by Matanock et al. (2018) who calculated that this figure is around 16 per year in the US alone, meaning that there are around 380 deaths a year.^14^ Therefore, parasite prevention measures and/or control policies in water bodies are urgently needed and sadly are currently limited to a few countries.

The infection usually begins with the inhalation of the trophozoite stage present in contaminated water bodies.^15,16^ When *N. fowleri* trophozoites reach the nasal area and are exposed to chemicals such as acetylcholine and neurotransmitters due to olfactory mucosal erosion, could initiate a chemotactic craving in them. Hence, triggering their locomotion along a chemical gradient toward the brain.^17^ Once it reaches the brain, it causes tissue destruction due to the action of different cysteine proteases released from the amoeba. This leads to the onset of symptoms, such as headache, fever, or nausea. Within a few days, additional severe symptoms appear including hallucinations and convulsions with a progressive tendency to coma followed by death of the patient.^18^

Currently, PAM remains a difficult disease to diagnose and, in most cases, the diagnosis is performed postmortem. The lack of awareness and the rapid progression of the disease, which makes an early detection of the pathogen necessary, are some of the issues that clinicians must face. At the present stage, diagnosis of PAM highly depends on awareness of this pathogen among the clinicians as well as expertise in both morphological and molecular detection of *N. fowleri.* Usually, a sample of cerebrospinal fluid is collected and if possible, the parasite is detected by the combination of three methods: morphological analysis of the amoeba from a wet cerebrospinal fluid preparation, differentiation tests for the different stages, and molecular identification by PCR.^19−23^

Regarding the treatment of PAM, the widely applied therapy involves a combination of amphotericin B and miltefosine and other drugs such as azoles in synergy, which have been shown to penetrate through the blood–brain barrier (BBB) and eliminate the amoebae at low concentrations.^9,24−28^ Recently, the in vivo activity of some novel molecules such as 4-aminomethylphenoxy-benzoxaborole AN3057 or antifungals such as Posaconazole has been demonstrated in murine models.^29,30^

On the other hand, studies have reported the biological activity of different cyano-substituted molecules in the last years, showing activity against several pathogenic organisms. For example, Quinoxalines have proven to be useful molecules for the development of new therapies against Tuberculosis and Chagas.^31^ Besides, other cyano-substituted molecules, such as 3,4-disubstituted pyrazolo[3,4-*d*]pyrimidine ribonucleosides and selenocyanate derivatives, have been reported to display in vitro activity against different protozoa such as *Leishmania* or *Trypanosoma cruzi*, respectively.^32,33^

Therefore, the aim of this study was to evaluate the antiamoebic activity of a collection of 46 synthetic cyanomethyl vinyl ethers provided by the *Instituto de Productos Naturales y Agrobiología* (IPNA), from the *Consejo Superior de Investigaciones Científicas* (CSIC). Moreover, those with the highest activity were used to determine the type of programmed cell death triggered in *N. fowleri*, as it has been shown in previous studies in our laboratory.^34,35^

## Results

### In Vitro Activity and Cytotoxicity Assays of Cyanomethyl Vinyl Ethers

Cyanomethyl vinyl ether conjugates were synthesized from the design of a novel organocatalytic multicomponent cyanovinylation of aldehydes in a previous study.^36^ From that study, 46 products were obtained and evaluated for their amoebicidal activity against the trophozoite form of two type strains of *N. fowleri* (ATCC 30808 and ATCC 30215). On the other hand, their toxicity was analyzed on murine macrophage-like cell line J774A.1 (ATCC TIB-67). Activity assays showed that, of the 46 products tested, seven of them were significantly active as shown in the table (Table 1). QOET-51, QOET-59, QOET-64, QOET-67, QOET-72, QOET-77, and QOET-79 were selected for their high activity (IC_50_) against the amoebae. From these seven products, QOET-77 showed the lowest IC_50_ in both strains and QOET-59 showed lowest toxicity, with a CC_50_ being higher than 800 μM, thus showing a good selectivity index.

**Table 1:** Activities (IC_50_) of Tested Cyanomethyl Vinyl Ethers Against the Two Strains of *Naegleria fowleri*, Indicating Also the Cytotoxic Concentration (CC_50_) in Murine Macrophages and Their Selectivity Index (SI) Against the Parasite^a^

Compound	*N. fowleri* ATCC® 30808^TM^ IC_50_ (μM)	*N. fowleri* ATCC® 30215^TM^ IC_50_ (μM)	Murine macrophages J774.A1 CC_50_ (μM)	S.I. ATCC® 30808^TM^	*N. fowleri* ATCC® 30808^TM^ IC_90_ (μM)
QOET-51	49.86 ± 3.04	68.51 ± 2.16	709.44 ± 67.13	14.23	172.79 ± 17.17
QOET-59	42.34 ± 6.44	44.45 ± 0.48	>864.86	>20	77.19 ± 11.76
QOET-64	38.81 ± 2.13	48.91 ± 0.81	>350.61	>9	83.44 ± 9.47
QOET-67	48.32 ± 10.48	121.48 ± 20.70	>325.41	>6	153.79 ± 15.26
QOET-72	47.71 ± 4.10	50.13 ± 4.90	>407.70	>7	184.69 ± 7.05
QOET-77	39.24 ± 6.30	42.74 ± 8.50	>393.59	>10	117.02 ± 26.06
QOET-79	43.28 ± 5.69	54.33 ± 7.40	>425.17	>10	119.05 ± 25.98
Amphotericin B	0.12 ± 0.03	0.16 ± 0.02	≥200	≥1652.89	0.35 ± 0.02
Miltefosine	38.74 ± 4.23	81.57 ± 7.23	127.89 ± 8.85	3.30	89.47 ± 17.37

a Moreover, the IC_90_s, used for programmed cell death assays in *N. fowleri* ATCC 30808, are indicated.

### Evaluation of PCD Induction in Treated Amoebae

#### Chromatin Condensation Assay

**Figure 1:**
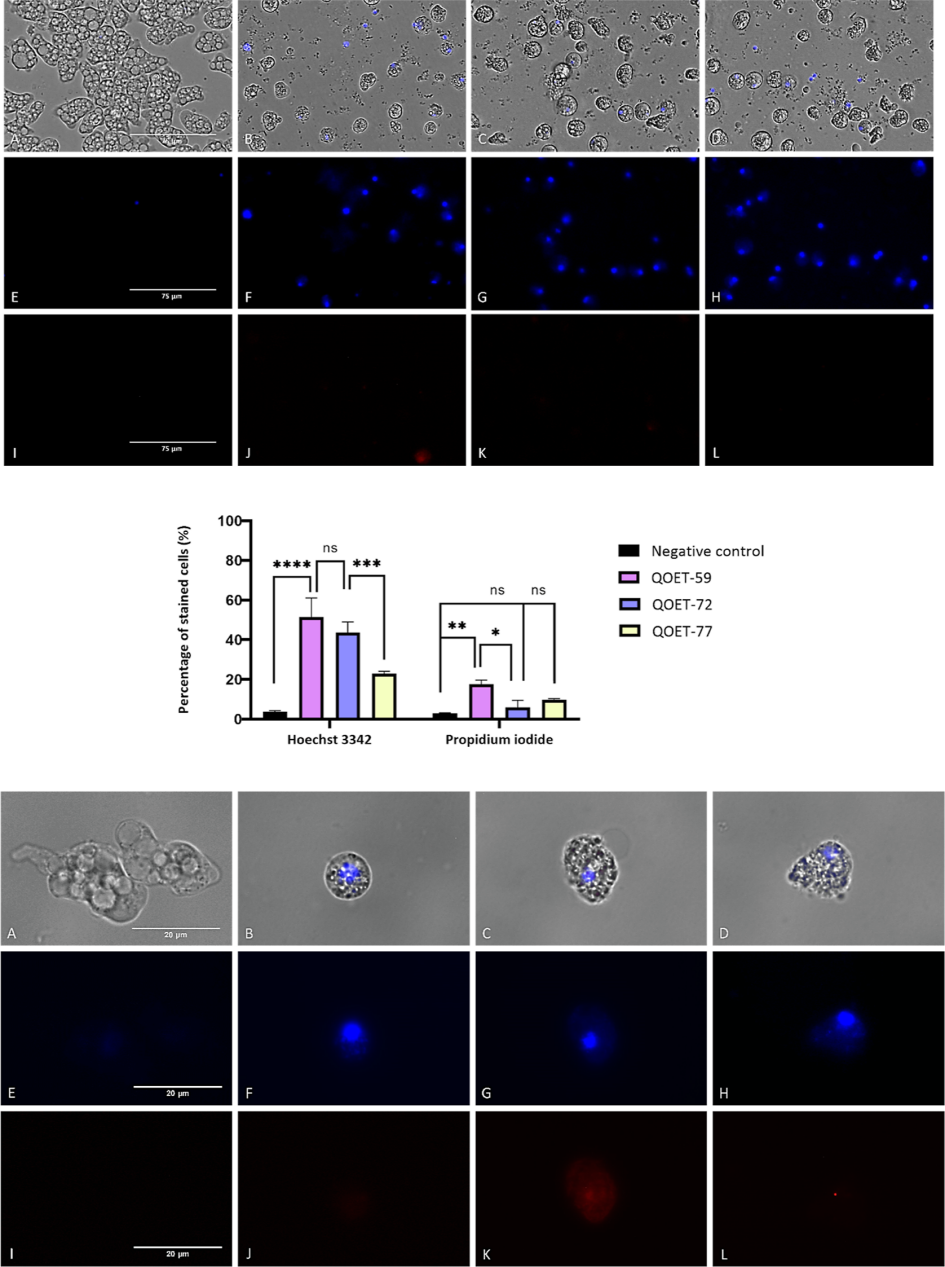
Chromatin condensation detection with a double-stain assay apoptosis detection kit (Hoechst 33342/PI). *Naegleria fowleri* trophozoites (ATCC 30808) incubated with IC_90_ of cyanomethyl vinyl ethers QOET-59 (B,F,J), QOET-72 (C,G,K), and QOET-77 (D,H,L) comparing with the negative control (A,E,I).Treated amoeba shows an intense blue stained nuclei (F–H) that correspond to the Hoechst fluorochrome whereas no fluorescence was observed in the control cells (E). Overlay channel (A–D); Hoechst channel (E–H) and propidium iodide channel (I–L). Images (1; ×40) (2; x100) are the representative of the cell population observed in the performed experiments. Images were obtained using a Discover Echo Inc. Revolution Microscope (Discover Echo, San Diego, CA).The bar graph includes the estimated percentage of stained cells. Differences between the values were assessed using an one-way analysis of variance (ANOVA). Data are presented as means ± SD (*N* = 3); ns: not significant, *p < 0.1; **p < 0.01; ***p < 0.001; ****p < 0.0001 significant differences when comparing treated cells to negative control. Mean percentage of stained cells for each assay was determined using the Discover Echo Inc. Revolution Microscope software. All the experiments were conducted in triplicate. (Images 1 scale bar: 75 μm and images 2 scale bar: 20 μm).

One characteristic event used to identify the apoptotic cells is chromatin condensation accompanied by DNA fragmentation.^37^ In treated amoebae with IC_90_ of QOET-59, QOET-72, and QOET-77, condensation was observed by the bright blue fluorescence in cells (Figure 1B–D,F–H). In addition, red fluorescence in treated amoebas with QOET-77 from propidium iodide was observed, indicating that cells are in a late apoptosis phase (Figure 1K). Furthermore, we observe the negative control where healthy amoebae do not show any fluorescence at all.

#### Disruption of Mitochondrial Membrane Potential

**Figure 2:**
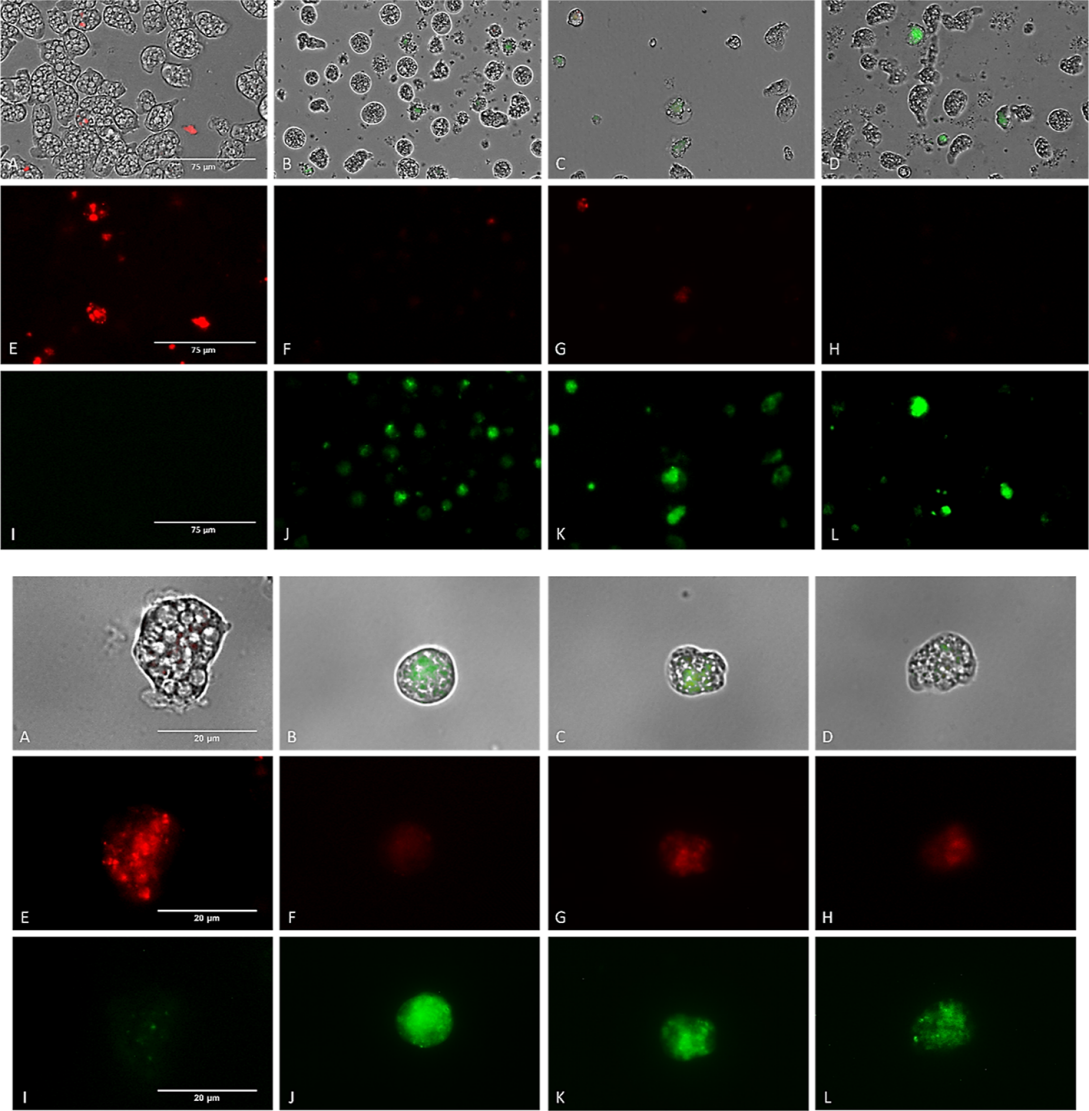
Analysis of mitochondrial membrane potential disruption in *Naegleria fowleri* (ATCC 30808). Trophozoites of *N. fowleri* (ATCC 30808) treated with QOET-59 (B,F,J), QOET-72 (C,G,K) and QOET-77 (D,H,L) using the reagent JC-1 Mitochondrial Membrane Potential Flow Cytometry Assay Kit and negative control (A,E,I). There was a disruption in the mitochondrial membrane potential of the treated cells (B–D, F–H, and J–L), which emit green fluorescence, while negative control (A,E,I) shows red fluorescence. Images (1; ×40) (2; ×100) are the representative of the cell population observed in the experiments and were captured using a Discover Echo Inc. Revolution Microscope (Discover Echo, San Diego, CA). (Images 1 scale bar: 75 μm and images 2 scale bar: 20 μm).

It is known that electron transport chain is responsible for generating mitochondrial transmembrane potential (ΔΨm). A vital function of this potential is the production of ATP via oxidative phosphorylation.^38^ Therefore, damage to any of the four major protein complexes would induce the changes in this potential and thus in ATP synthesis. This event could be demonstrated using the JC-1 reagent, which emits fluorescence at two different wavelengths. Under normal conditions, the reagent internalizes into the mitochondria in the form of J-aggregates and emits red fluorescence (Figure 2 E). In contrast, in treated amoebae, in which cell damage is present, resulting in a loss of mitochondrial membrane potential (ΔΨm), the reagent is spread as a monomer showing green fluorescence (Figure 2B–D,J–L).

#### Determination of ATP Levels

**Figure 3:**
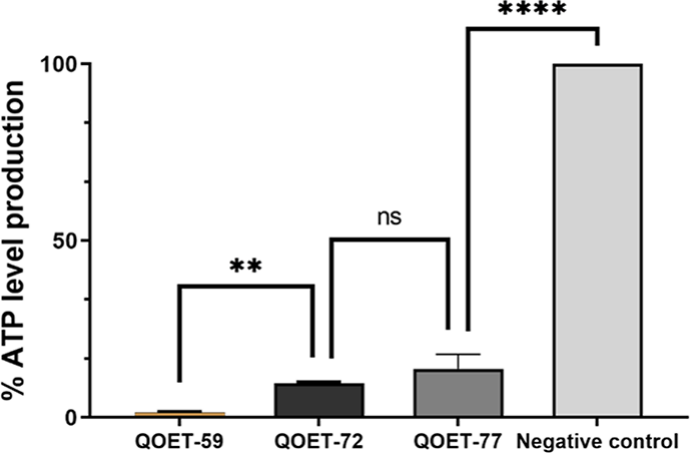
ATP production in percentages relative to the negative control (healthy trophozoites) after 24-h incubation of amoebae with IC90 of the molecules, using the CellTiter-Glo Luminescent Cell Viability Assay. Statistics show a decrease in ATP levels of 98.49% (QOET-59), 90.25% (QOET-72), and 86.28% (QOET-77) compared to the negative control. Differences between values were assessed by the one-way analysis of variance (ANOVA). Data are presented as mean ± SD (*N* = 3); ns: not significant, **p < 0.01 and ****p < 0.0001 are significant differences when comparing the different mean values.

To corroborate the mitochondrial damage in treated amoebae, the levels of ATP produced after 24 h were determined. The results showed a decrease in the production of ATP. Compared to the negative control, QOET-59, QOET-72, and QOET-77 decreased the ATP levels by 98.49, 90.25, and 86.28%, respectively (Figure 3).

#### Permeability of Plasma Membrane

**Figure 4:**
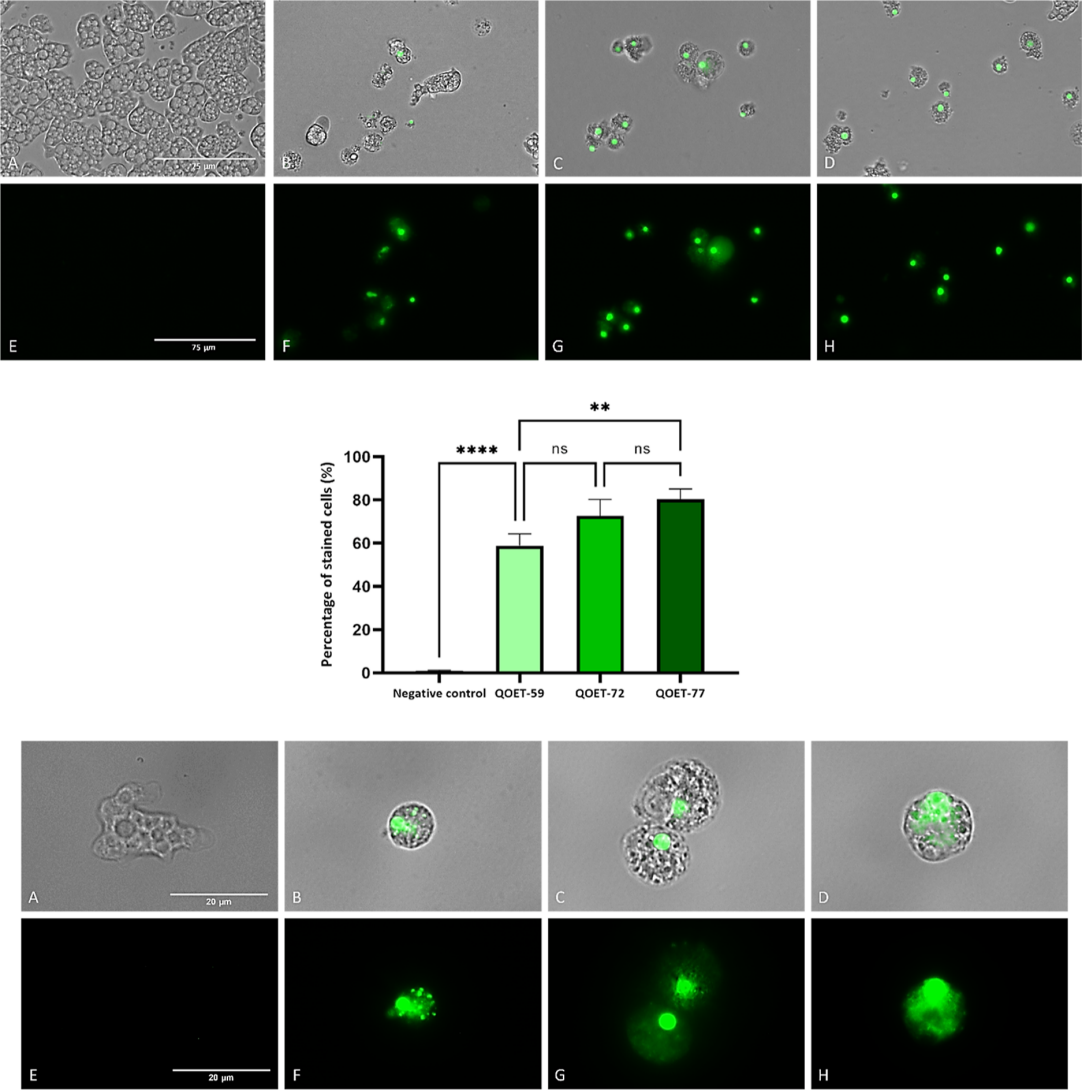
Evaluation of the integrity of the plasma membrane with the SYTOX Green viability assay. Treated *Naegleria fowleri* (ATCC 30808) trophozoites with QOET-59 (B and F), QOET-72 (C and G), and QOET-77 (D and H) and negative control (A and E). Treated cells (B–D and F–H) show green fluorescence, and negative control (A and E) shows no fluorescence. Images (1; ×40) (2; x100) are the representative of the cell population observed in the experiments and were captured using a Discover Echo Inc. Revolution Microscope (Discover Echo, San Diego, CA). The bar graph includes the estimated percentage of stained cells. Differences between the values were assessed using the one-way analysis of variance (ANOVA). Data are presented as means ± SD (*N* = 3); ns: not significant, **p < 0.01; ****p < 0.0001 significant differences when comparing treated cells to negative control. Mean percentage of stained cells for each assays was determined using the Discover Echo Inc. Revolution Microscope software. All the experiments were conducted in triplicate. (Images 1 scale bar: 75 μm and images 2 scale bar: 20 μm).

The SYTOX Green reagent was used to demonstrate the alterations in the permeability of plasma membrane. Under normal conditions, the cell membrane is impermeable to the reagent, and hence, no fluorescence will be emitted (Figure 4E). In contrast, when cells were treated with the compounds, damage was observed, causing an increase in the permeability of the membrane, allowing it to internalize into the cell and bind to nucleic acids, emitting an intense green fluorescence (Figure 4B–D,F–H).

#### Analysis of Oxidative Stress

**Figure 5:**
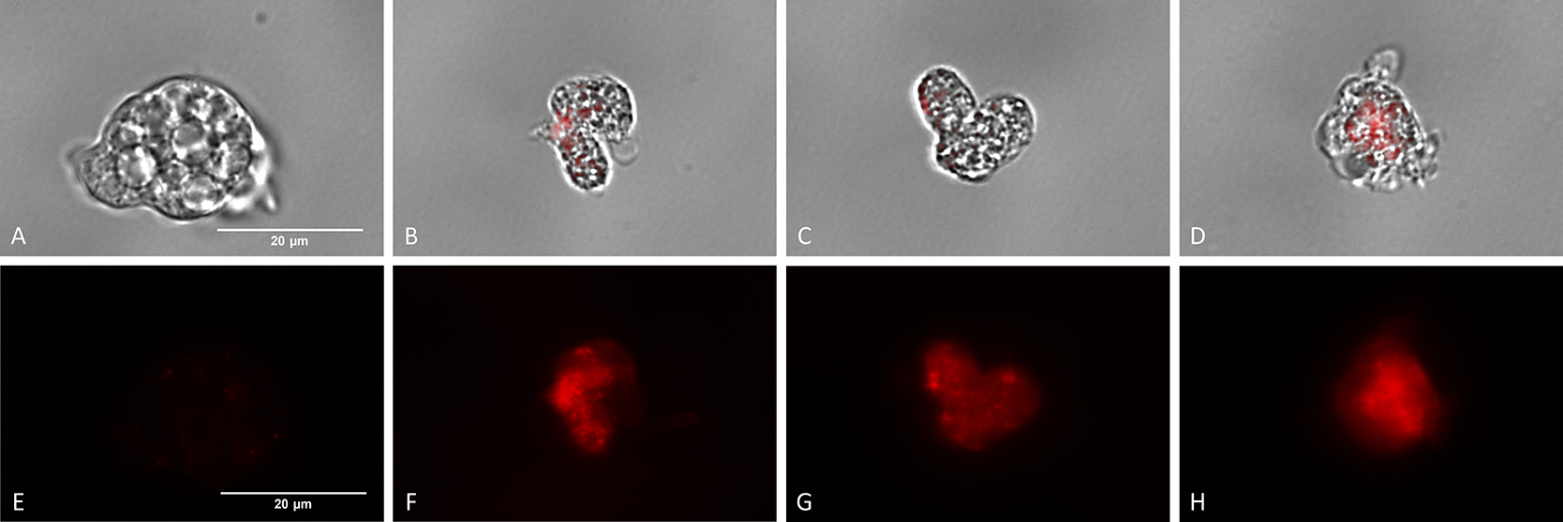
ROS detection in treated amoebae using CellROX. *Naegleria fowleri* trophozoites treated with the IC_90_ of QOET-59 (B and F), QOET-72 (C and G), and QOET-77 (D and H) during 24 h show an intense red fluorescence due to the increase of the intracellular ROS generation. Negative control (A and B). Images (1; ×40) (2; ×100) are representative of the cell population observed in the experiments and were captured using a Discover Echo Inc. Revolution Microscope (Discover Echo, San Diego, CA). The bar graph includes the estimated percentage of stained cells. Differences between the values were assessed using the one-way analysis of variance (ANOVA). Data are presented as means ± SD (*N* = 3); ns: not significant, *p < 0.1; **p < 0.01; ***p < 0.001; ****p < 0.0001 significant differences when comparing treated cells to negative control. Mean percentage of stained cells for each assays was determined using the Discover Echo Inc. Revolution Microscope software. All the experiments were conducted in triplicate. (Images 1 scale bar: 75 μm and images 2 scale bar: 20 μm).

To demonstrate the oxidative stress generated in treated cells, the presence of reactive oxygen species generated was measured. For this reason, CellROX Deep Red kit was used, which emits red fluorescence upon binding in the cytoplasm of the cell when encountering various molecules formed from oxygen such as superoxide (O2-), hydroxyl (OH), or hydrogen peroxide, among others. All selected compounds caused an increment of ROS levels in treated amoebae (Figure 5B–D,F–H).

#### Depolymerization of Actin Filaments

Regarding the cytoskeleton structure, this is mainly made up of actin filaments, which grows by their polymerization. In this assay, phalloidin-TRITC, which binds to these filaments, was used to demonstrate the integrity of the cytoskeleton. Under optimal conditions, the reagent binds to the filaments (F-actin) emitting red fluorescence, which is more intense in certain regions related to movement and/or phagocytosis. On the other hand, when cell damage occurs, there is an overall decrease in fluorescence due to depolymerization of the filaments and actin cytoskeleton being much less organized.

**Figure 6:**
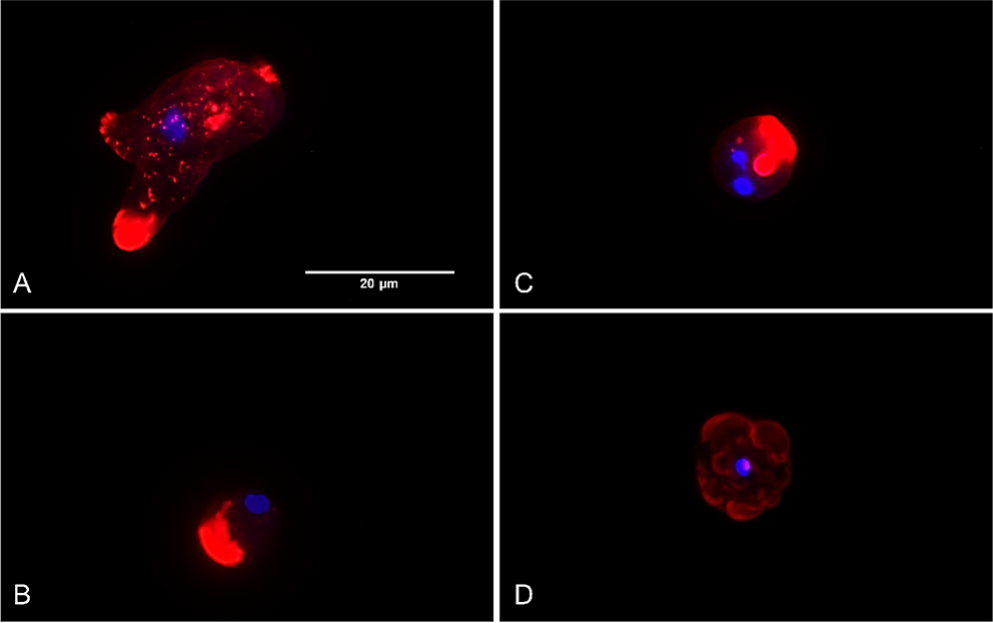
presence of actin filaments using phalloidin-TRITC. Z-stack imaging of treated amoebae with the IC_90_ of QOET-59 (B), QOET-72 (C), QOET-77 (D), and negative control (A) showing red (actin filaments) and blue fluorescence (condensed chromatin in the nucleus). Actin filaments (F-actin) in nontreated cells emitted red fluorescence and a normal conformation of the actin network. In treated cells, a decreased level of fluorescence was observed as well as a less organized actin network (B–D) when compared to the negative control (A). Images are representative of the cell population observed in the performed experiments. Images were obtained using a confocal microscope Leica DMI4000 B with LAS X software, a 405 nm laser and 532 nm laser, and Leica HCX PL Apo 63x Oil Objective were used (Scale bar: 20 μm).

All cells treated with the compounds showed a dramatical modification of their morphology when compared to the control. In all treatment cases (Figure 6B–D), trophozoites emitted a lower red fluorescence compared to healthy cells (Figure 6A), which showed a normal conformation of the actin network. Moreover, QOET-59- and QOET-72-treated cells emitted a bright red fluorescence region related to actin depolymerization. In the case of QOET-77, the observed damage was higher when compared to the control and only low red fluorescence was detected (Figure 6D).

#### In Vitro Activity of Cyanomethyl Vinyl Ethers Against the Cyst Stage

The activity of QOET-59, QOET-72, and QOET-77 cyanomethyl vinyl ethers against the cyst stage of *N. fowleri* (ATCC 30808) was evaluated. In general, the evaluated molecules were active against the cyst stage at IC_50_ twofold higher (Table 2) than the IC_50_s for the trophozoite stage (Table 1). In addition, the compound with the highest activity was QOET-59 (IC_50_: 93.56 ± 17.47 μM) although its activity was still not as high as the activity obtained for the trophozoites (IC_50_: 44.45 ± 0.48 μM) (see Table 3).

**Table 2:** Cysticidal Activity of the QOETs That Induced PCD Events in *Naegleria fowleri* ATCC 30808

compound	*N. fowleri* ATCC® 30808^TM^ IC_50_ (μM)
QOET-59	93.56 ± 17.47
QOET-72	130.44 ± 22.79
QOET-77	101.62 ± 7.01

**Table 3:**
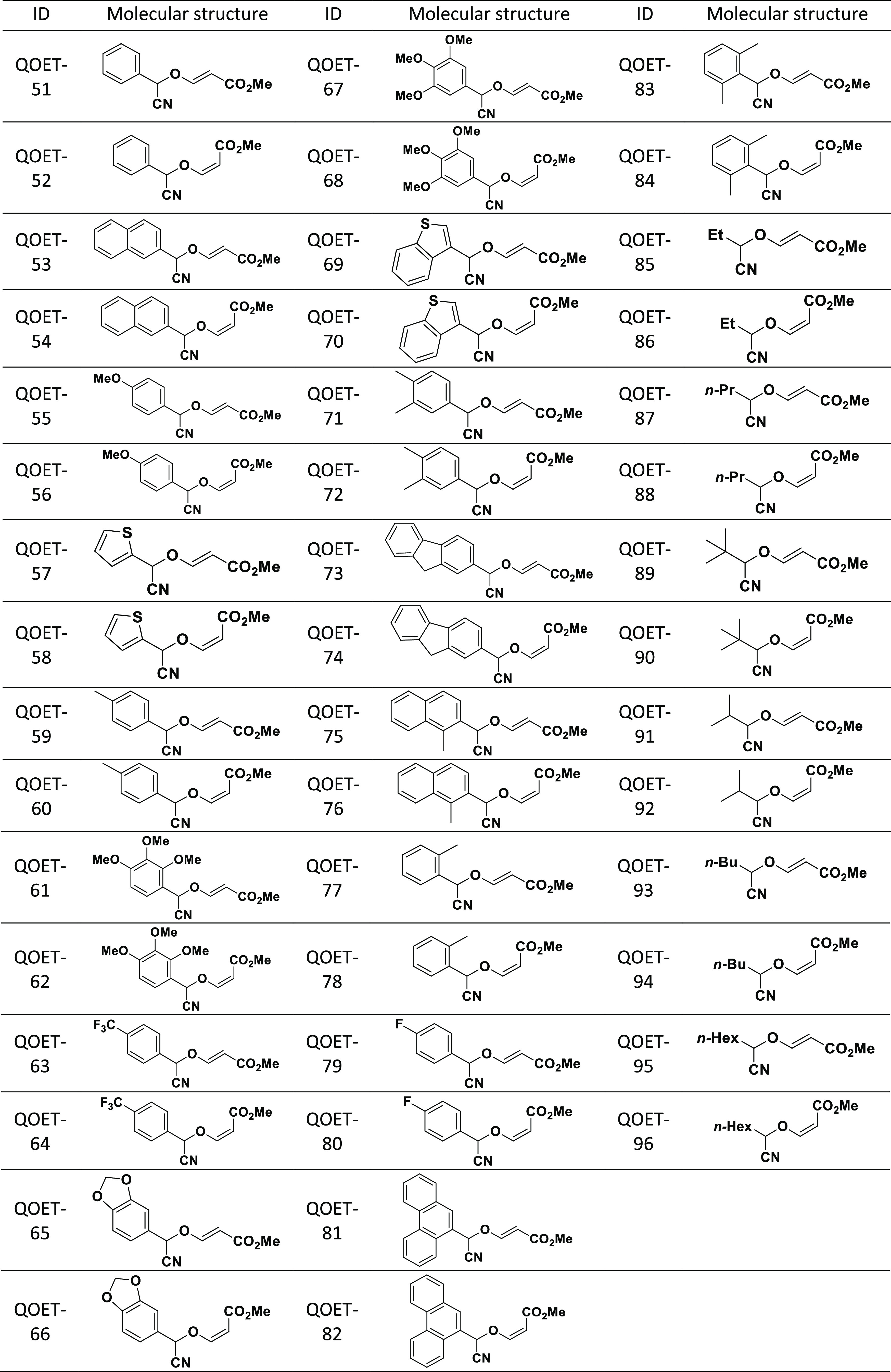
Chemical Structure of Cyanomethyl Vinyl Ethers

## Discussion

The aim of this study was to determine the activity of 46 novel cyanomethyl vinyl ethers against *N. fowleri.* In addition, seven of the tested compounds were active against the trophozoite stage of these amoebae (Table 1). Furthermore, these active compounds showed selectivity indexes higher than the one obtained for the reference drug miltefosine. In addition, the cysticidal activity of these three products was also demonstrated (QOET-59, QOET-72, and QOET-77). Moreover, the higher activity observed in the case of these products could be due to a longer carbon chain (hexyl groups) and hence their higher lipophilic character in these three compounds when compared to the other 43 evaluated ones.

After that, PCD induction assays were performed with QOET-59-, QOET-72-, and QOET-77-treated amoebae. All compounds gave evidence of PCD traits which were identified using reagents and fluorescence microscopy, as previously described in our laboratory for other drugs^39^ and also by other studies from Cárdenas-Zúñiga et al. (2017).^40^

Among the PCD-related events identified in our study, the selected cyanomethyl vinyl ethers were able to produce chromatin condensation, increased plasma membrane permeability, oxidative stress generation, changes in the mitochondrial membrane potential, and, consequently, a decrease in ATP production. When novel therapeutic agents are seek, the type of cell death is important in order to prevent the inflammatory processes of necrotic cell death process.^41,42^ In the near future, these molecules could be further exploited for PAM therapy due to their lipophilicity and low molecular weight (less than 500 Da), which could facilitate the penetration of the blood–brain barrier.^43^

In addition, an assay to show the arrangement of actin filaments as the main component of *N. fowleri* cytoskeleton using Z-Stack imaging was carried out using phalloidin-TRITC.^44,45^ In treated amoebae, a disassembly of the cytoskeleton was shown (Figure 6), leading to the alterations in cell shape and clusters of depolymerized actin filaments in the located areas of these cells (Figure 6B,C). Therefore, further studies using these compounds could be focused on elucidating whether the amoebic cytoskeleton is a direct target for them.^46−48^

## Conclusions

In conclusion, the selected compounds (QOET-59, QOET-72, and QOET-77) showed high selectivity against two strains of *N. fowleri*, inducing apoptotic cell death processes. Therefore, this novel pool of synthesized products may generate a new source of therapeutic agents against PAM.

## Material and Methods

### Procedure of Synthesis of Cyanomethyl Vinyl Ethers

In this study, 46 cyanomethyl vinyl ethers, whose chemical synthesis has already been described and published by Delgado-Hernández et al. (2021), were used. Amphotericin B (Sigma-Aldrich, Madrid, Spain) and miltefosine (Cayman Chemicals, Vitro SA, Madrid, Spain) were used as positive control drugs against *N. fowleri*.

#### Amoebae and Cell Culture

For this study, two type strains of *N. fowleri* (ATCC 30808 and ATCC 30215) were used to perform in vitro assays, with the aim of obtaining activity in these strains as they have shown variable susceptibility to the drugs by different genotypes of this species.^49^*N. fowleri* ATCC 30808 Strain KUL origin from Belgium and *N. fowleri* ATCC 30215 Strain Nf69 was geographically isolated from Australia and South Africa, both obtained from clinical samples. The strains in trophozoite form were grown under axenic conditions in 2% (w/v) Bactocasitone medium, supplemented with 10% (v/v) heat-inactivated fetal bovine serum (FBS), 0.5 mg/mL of streptomycin sulfate (Sigma-Aldrich, Madrid, Spain) and 0.3 μg/mL penicillin G (sodium salt) (Sigma-Aldrich, Madrid, Spain). The trophozoites in the medium were incubated at 37 °C facilitating their multiplication. For the formation of cysts, trophozoites of *N. fowleri* ATCC 30808 strain were cultured on MYAS liquid medium at 28 °C with slight agitation in an orbital shaker for 10 days, as previously described.^50^ All *N. fowleri* strains were cultured in a biosafety level 3 (BSL-3) laboratory facility at the Instituto Universitario de Enfermedades Tropicales y Salud Pública de Canarias, University of La Laguna, in accordance with the Spanish governments’ biosafety guidelines for this pathogen, which considers exposure to aerosols or droplets formed on mucous membranes of the eyes, nose, or mouth to trophozoites as a potential hazard when working with free-living amoebae cultures.

For cytotoxicity assays, a murine cell line macrophage J774A.1 (ATCC TIB-67) was used, maintained in the Dulbecco’s modified Eagle’s medium (DMEM, w/v) supplemented with 10% (v/v) FBS and 10 μg/mL gentamicin (Sigma-Aldrich, Madrid, Spain), incubated at 37 °C in a 5% CO_2_ atmosphere.

#### In Vitro Activity Against Trophozoite Stage of *N. fowleri*

In vitro activity assays against *N. fowleri* trophozoites were performed according to the protocol using a colorimetric assay based on the cell viability reagent alamarBlue.^39^ After 48 h of incubation, the fluorescence of the plates was analyzed using the EnSpire Multimode Plate Reader (PerkinElmer, Madrid, Spain). The results obtained for inhibitory concentration 50 (IC_50_) and 90 (IC_90_) were calculated using GraphPad Prism 9 software by nonlinear regression analysis.

#### In Vitro Cytotoxicity Against Murine Macrophages Cell Line

In this assay, a murine macrophage cell line J774A.1 (ATCC TIB-67) was used. Serial dilutions of the compounds were added and, subsequently, the alamarBlue reagent as described in previous studies^51^ was also placed. Once the plates were read on the EnSpire Multimode Plate Reader (PerkinElmer, Madrid, Spain), the data were analyzed to obtain the cytotoxic concentration 50 (CC_50_). This CC_50_, together with the activity, was used to obtain the selectivity index for *N. fowleri.*

#### Programmed Cell Death (PCD) Induction in *N. fowleri*

To evaluate the mechanisms of action of the compounds at the cellular level, a series of commercial kits were used according to the manufacturer’s instructions. To carry out the assays, the *N. fowleri* ATCC 30808 culture was previously incubated with the IC_90_ of the compounds evaluated for 24 h. Nontreated *N. fowleri* trophozoites were used as a negative control. After this time, the reagents for each kit were added, and the effect was observed with a Discover Echo Inc. Revolution Microscope (Discover Echo, San Diego, CA), analyzing the fluorescence using an EnSpire Multimode Plate Reader (PerkinElmer, Madrid, Spain). Five images were processed each time at 40x and 100x objective lenses.

#### Induction of DNA Condensation

A double-stain apoptosis detection kit (Hoechst 33342/PI) (Life Technologies, Madrid, Spain) was used to evaluate DNA condensation based on the fluorescent detection for chromatin compaction in apoptotic cells, and the assay offers a rapid and convenient apoptosis detection. As soon as the amoebae have been incubated for 24 h with the active compound’s IC_90_, Hoechst and propidium iodide (PI) are added at a final concentration of 5 and 1 μg/mL, respectively. Fluorescence should be observed after 15 min of incubation in the dark. In apoptotic cells, Hoechst 33342, a dye for blue fluorescence, stains the condensed chromatin more intensely than that in normal cells. PI emits red fluorescence (maximum excitation and emission wavelengths when bound to DNA of 535 and 617 nm, respectively), which can only be emitted through dead cells. These assays could be demonstrated using the images obtained on a Discover Echo Inc. Revolution Microscope (Discover Echo, San Diego, CA).

#### Analysis of Mitochondrial Function Disruption

To determine the potential of the mitochondrial membrane was use the dye JC-1 (1,1′,3,3′-tetraethyl-5,5′,6,6′-tetrachloroimidacarbocyanine iodide) (Cayman Chemicals Vitro SA, Madrid, España). Once the amoebae were incubated for 24 h with the IC_90_ of the active compounds, 10 μL of JC-1 was added. The plate was incubated in the dark for 20 min and the emitted fluorescence was observed in a Discover Echo Inc. Revolution Microscope (Discover Echo, San Diego, CA) and quantified using EnSpire Multimode Plate Reader (PerkinElmer, Madrid, Spain). The reagent fluoresces at two different wavelengths depending on the membrane potential. Under normal conditions, the monomers bind together to form J-aggregates (dimers) emitting red fluorescence at 595 nm. In contrast, in treated amoebae, where cell damage is present, where there is a loss of mitochondrial membrane potential (ΔΨm), the reagent is dispersed as a monomer and shows green fluorescence at 535 nm.

#### Determination of ATP Levels

In order to measure the amount of ATP produced, Cell Titter-GLO Luminescent Cell Viability assay (Promega Biotech Ibérica, Madrid, Spain) was used. With this kit, it is possible to compare the ATP production of treated cells to that of nontreated (negative control). ATP levels were evaluated by luminescent analysis using an EnSpire Multimode Plate Reader (Perkin Elmer, Madrid, Spain) following incubation with IC_90_ of selected compounds at 37 °C for 24 h.

#### Evaluation of Plasmatic Membrane Permeability

The permeability of the plasma membrane can be analyzed using a green fluorescent dye called SYTOX Green (Life Technologies, Madrid, Spain). After the amoebae are incubated for 24 h with the IC_90_ of cyanomethyl vinyl ethers, SYTOX Green was added to a final concentration of 1 μM and after 15 min of incubation, cells were observed using Discover Echo Inc. Revolution Microscope (Discover Echo, San Diego, CA). The plasma membrane of the damaged cells will become permeable, giving the reagent the ability to enter through it. When inside the cell, it binds to the DNA and increases its fluorescence more than 500-fold, emitting green fluorescence at 523 nm.

### Generation of Oxidative Stress

In this assay, the oxidative stress produced in the pathogen by the generation of oxygen free radicals is evaluated. The CellROX Deep Red kit (Invitrogen, Thermo Fisher Scientific, Madrid, Spain) was used in amoebae incubated for 24 h with the IC_90_ of the compounds to be tested. The reagent was used at a final concentration of 5 μM and incubated for 30 min. After this time, the action of the reagent was observed using the Discover Echo Inc. Revolution Microscope (Discover Echo, San Diego, CA). As a result of the overproduction of reactive oxygen species (ROS), this dye has the ability to penetrate the cells and emit red fluorescence at a wavelength of ∼644/665 nm.

#### Evaluation of the Cytoskeleton by Actin Depolymerization

The disassembly of cytoskeleton in treated amoebae can be analyzed by the red fluorescence emitted by phalloidin-TRITC when it binds to actin filaments. On the other hand, DAPI reagent was used which, in turn, binds to DNA and emits blue fluorescence, as already described in *Acanthamoeba* genus.^52^ In this assay, amoebae were incubated with the IC_90_ of compounds. A 24-h incubation period was followed by the fixation of the cells with formaldehyde on a coverslip and the addition of 0.1% Triton before the addition of phalloidin-tetramethylrhodamine B isothiocyanate (phalloidin-TRITC; Sigma-Aldrich, Madrid, Spain) for another 30-min period. Finally, after incubation, the sample was washed with PBS. Cells were examined by Z-stack with a 100x objective of the EVOS FL Cell Imaging System AMF4300 (Life Technologies, Madrid, Spain).

#### In Vitro Activity Against Cyst Stage of *N. fowleri*

Once mature cysts from the culture were obtained, the activity plate was made with the compounds of interest. For this, serial dilutions of the compounds together with the Bactocasitone medium were added (50 μL) to a 96-well microtiter plate (Thermo Fisher Scientific, Madrid, Spain). This is followed by removing the medium and adding it again, in order to excyst and confirm the viability of the cysts. After that, another 50 μL of *N. fowleri* cysts were added at a concentration of 2 × 10^5^ cells/ml. At last, 10% alamarBlue reagent was placed in each well, reducing from nonfluorescent resazurin to fluorescent resorufin when trophozoites emerge from cyst stage.^50^ Then, 24 h after adding the reagent, the fluorescence was analyzed on the EnSpire Multimode Plate Reader (PerkinElmer, Madrid, Spain) to obtain inhibitory concentration 50 (IC_50_) value.

## Data Analysis

All assays were performed in triplicate and the results were defined as the mean obtained from the three experiments. Statistical analyses and inhibition curves obtained were performed using the GraphPad Prism 9.0 software (GraphPad Software; CA; USA). Differences between values were estimated by the one-way analysis of variance (ANOVA). Data are presented as mean ± standard deviation, and *p*-value <0.05 was considered statistically significant.

## Abbreviations

ANOVA: one-way analysis of variance
ATCC: American Type Culture Collection
BBB: blood–brain barrier
BSL-3: biosafety level 3
CC_50_: cytotoxicity concentration
CNS: central nervous system
CSIC: Consejo Superior de Investigaciones Científicas
DAPI: 4 ′,6-diamidino-2-phenylindole
DMEM: Dulbecco’s modified Eagle’s medium
DNA: deoxyribonucleic acid
FBS: fetal bovine serum
GFP: green fluorescent protein
IC_50_: inhibition concentration 50
IC_90_: inhibition concentration 90
IPNA: Instituto de Productos Naturales y Agrobiología
JC-1: 5,5′,6,6′-tetrachloro-1,1′,3,3′-tetraethylbenzimidazolocarbocyanine iodide
MYAS: malt extract, yeast extract, and amoebae saline
PAM: primary amoebic meningoencephalitis
PCD: programmed cell death
PCR: polymerase chain reaction
PI: propidium iodide
RFP: red fluorescent protein
ROS: reactive oxygen species
SI: selectivity index
ΔΨm: mitochondrial transmembrane potential
